# Posterior capsular opacification comparison between morphology and objective visual function

**DOI:** 10.1186/s12886-019-1051-z

**Published:** 2019-02-04

**Authors:** Chengzhe Lu, Shasha Yu, Hui Song, Yun Zhao, Shiyong Xie, Xin Tang, Xiaoyong Yuan

**Affiliations:** 1 Tianjin Eye Hospital, No.4 Gansu Road, Heping District, Tianjin, 300020 China; 2 Tianjin Eye Institute, Tianjin, China; 3 Tianjin Key Laboratory of Ophthalmology and Visual Science, Tianjin, China; 4 Clinical College of Ophthalmology, Tianjin Medical University, Tianjin, 300020 China

**Keywords:** PCO morphology, Severity, Objective visual function, Objective scatter index (OSI), Optical quality analysis system values (OVs)

## Abstract

**Background:**

To compare the influence of posterior capsule opacification (PCO) morphology and severity on intraocular stray light and visual function with different levels of contrast.

**Methods:**

Forty-five patients diagnosed with PCO were included in this prospective consecutive case series. The Optical Quality Analysis System II (OQAS II) was adopted to assess the objective visual function including objective scatter index (OSI) and optical quality analysis system values (OVs) with 100, 20, and 9% contrast. RTVue-100 OCT was used to evaluate the PCO morphology and severity. Comparisons among visual function, morphology, and severity between pear type and fibrosis PCO were performed. The correlations among the PCO morphology, severity, OSI, and OVs were also determined.

**Results:**

There was a significant correlation between increased OSI and decreased visual acuity in PCO patients before laser capsulotomy. The changes of OSI were also correlated with the PCO area for the 3 mm IOL optic region (r = 0.43, *p* = 0.02). The OSI was significantly higher in pear type PCO when compared with fibrosis PCO (Z = − 4.06, *p* ≤ 0.001). In addition, the increased OSI in pear type PCO was significantly correlated with the 100% OVs and the 20% OVs but not with the 9% OVs. In fibrosis PCO, OSI was only correlated with the 100% OVs and the 20% OVs pre-YAG.

**Conclusions:**

OSI and OVs could objectively indicate the visual function impairment in PCO patients. Effects of PCO on light scattering and on objective visual function might be explained by the variations of morphology and severity.

## Background

Posterior capsule opacification is still the major complication after cataract surgery, which impairs the visual function of 28% of patients 5 years after IOL implantation [1]. Laser capsulotomy, the most common method, has been used to remove the opacification of the posterior capsule. The decision to perform laser capsulotomy surgery commonly involves decreased visual acuity and visual disturbances. In detail, decreased visual function has two distinct functional domains: visual acuity (VA) and contrast sensitivity (CS) assessed by the small angle domain as well as stray light assessed by the large angle domain [2]. It was reported that approximately 10% of intraocular light is scattered in the young healthy eye, but the number increases considerably in those over 50 years old [3]. In PCO patients, it was proven that forward light scatter was the most sensitive factor in PCO assessment; less than 1% of central PCO proved the increase in forward light scatter with the increase in PCO percentage, followed by the ETDRS visual acuity (78%) and the contrast sensitivity (ranged from 38 to 51%) [4].

In previous studies, C-quant was used to assess the forward light scatter. However, this approach was based on the subjective compensation comparison method [5–7] and was patient-dependent and time consuming [8]. Until now, the Optical Quality Analysis System II (OQAS II) based on the double-pass technique has enabled the objective evaluation of the visual system, including the measurement of forward light scattering, measurement with the objective scatter index (OSI) and the visual function at different levels of contrasts [9–12]. Studies have supported the repeatability and accuracy of OQAS-II application [13, 14].

Since previous studies have emphasized the influence of PCO morphology and severity on visual function, pear type and fibrosis PCO affect visual function, including visual acuity and contrast sensitivity, differently [8, 11, 15]. In vivo studies on the influence of PCO morphology and severity on objectively measured visual function are rare [16]. Furthermore, OCT as the backward light scattering method has been suggested as a method to evaluate the morphology of PCO [17].

Therefore, we combined the forward scatter method, OQAS-II, to assess the objective visual function and the backward scatter method, OCT, to assess the morphology of PCO. We aimed to compare the influence of different kinds of PCO morphology and severity on intraocular stray light and on visual function with different levels of contrast. We believe that objective visual function evaluation combined with morphology assessment plays an important role in PCO prevention and treatment.

## Methods

### Patients

This prospective study recruited posterior capsule patients in Tianjin Eye Hospital. The study was reviewed and approved by the Research Review Broad of Tianjin Eye Hospital. Informed consent from each patient was collected before the examination and surgery, and the study strictly adhered to the tenets of the Declaration of Helsinki (1989). Patients with corneal opacity, glaucoma, trauma, complicated ocular surgery, and severe systemic disease were excluded from the study. All the surgeries were performed by one expert doctor. Routine ocular examinations including visual acuity VA (corrected visual acuity, CDVA), intraocular pressure, auto-refractometer examination, slip lamp examination and fundus examination were done before surgery.

### Examinations

VA measured with the logarithmic visual acuity chart and converted to the logarithm of the minimum angle of resolution (logMAR) values for analysis.

OQAS II (Visiomtrics. Inc., Spanish) was adopted to access the quality of the visual system, the measurements including objective scatter index (OSI) and the optical quality analysis system values (OVs). The pupil diameter setting was 4 mm. Patients’ refractive errors were corrected by setting the real refractive state in the equipment before measurement. Patients were told to blink before measurement to reduce the influence of tear film. OSI was an objective quantification of the intraocular scattered light, which was defined as the ratio between the integrated light in the periphery and in the surroundings of the central peak of the double pass image. The central area was a circle with a radius of 1 min of arc, and the peripheral was a ring set between 12 and 20 min of arc. The OSI for normal eyes is approximately 1, and values over 5 represent highly scattered systems [9]. OVs referred to the objective visual acuity, corresponded to the modulation transfer function (MTF) values, and described the optical quality of the eye in at three contrast conditions, including 100% OVs, 20% OVs and 9% OVs. In detail, the 100% OVs were related to the MTF cutoff frequency, which was the MTF cutoff frequency divided by 30 cycles/degree. Therefore, the 100% OVs reflected the visual acuity with 100% contrast without the influence of the retina and neurons. The 20% OVs and 100% OVs were calculated in the same way from smaller frequencies, which were related to the 0.05 and 0.1 MTF value [18].

**Fig. 1:**
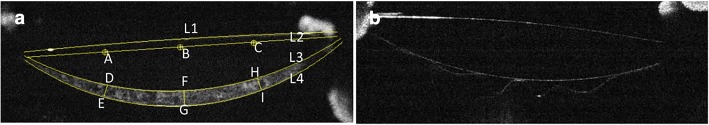
Illustration of PCO evaluation using RTVue-100 OCT. **a** Assessment of PCO with cross-sectional image. L refers to line. L1, L2, L3, and L4 represent the anterior surface of IOL, the horizontal diameter of the IOL optic region, the posterior surface of IOL, and the posterior capsule, respectively. Distance from point A to C refers to the 3-mm IOL optic region, and point B is the center. Distances from point D to E, H to I, and F to G are the PCO thickness at the 3-mm IOL optic region and the central optic region. The IOL-posterior capsular space is the region between line 3 and line 4. The region around point DFHIGE is the PCO area at the 3-mm IOL optic region. **b** Pseudophakic eye with a clear posterior capsule

RTVue-100 OCT (RTVue-100, Optovue Inc., Fremont, CA) was used to evaluate the area, thickness and density of PCO with cross sectional images on the horizontal and vertical meridian at 3 mm IOL optic region. After pupil dilation, the corneal anterior module long adaptor lens was installed to the detecting probe. Patients fixated on the front red indicator light using the other eye. The cornea cross line mode was used to take the images of the PCO on the vertical and horizontal meridians. The images were then transferred to a personal computer for further analysis using Image-Pro Plus software 6.0. The area, thickness and density of the opacification at the 3 mm optic zone were measured. Similar method was used to value opacification area, thickness and density at 5 mm optic zone. Values of the vertical and horizontal meridians were then averaged for further analysis (details in Fig. 1a).

VA and OQASII were measured within 10 to 30 min after the laser capsulotomy.

### Statistical analysis

All the data were recorded in Excel and were transferred to IBM SPSS Statistics 23.0 for analysis. The distribution of all the data was accessed using the Kolmogorov-Smirnov test before analysis. The CDVA and OSI before and after laser capsulotomy were compared using Wilcoxon matched paired test. The nonparametric Spearman correlation test was adopted to evaluate the relationship between variables. Comparisons of morphology, CDVA, OSI and OVs between pear type and fibrosis PCO were analyzed using the Mann-Whitney U test. *P*-values less than 0.05 were considered statistically significant.

## Results

The study included 48 eyes from 45 patients, including 28 eyes with pear type PCO, 14 eyes with fibrosis PCO, and 6 eyes with mixture PCO. The mixture type was excluded from further analysis. All the patients included completed the pre-Yttrium Aluminum Garnet (YAG) OQASII measurement, and 34 eyes completed the post-YAG OQASII measurement (26 eyes with pear type PCO and 8 eyes with fibrosis PCO). The average age was 69.17 + 10.93 years. PCO time was 43.24 + 30.67 months (ranges: 10–132 months).

### Comparison of VA and OSI before and after laser capsulotomy

**Fig. 2:**
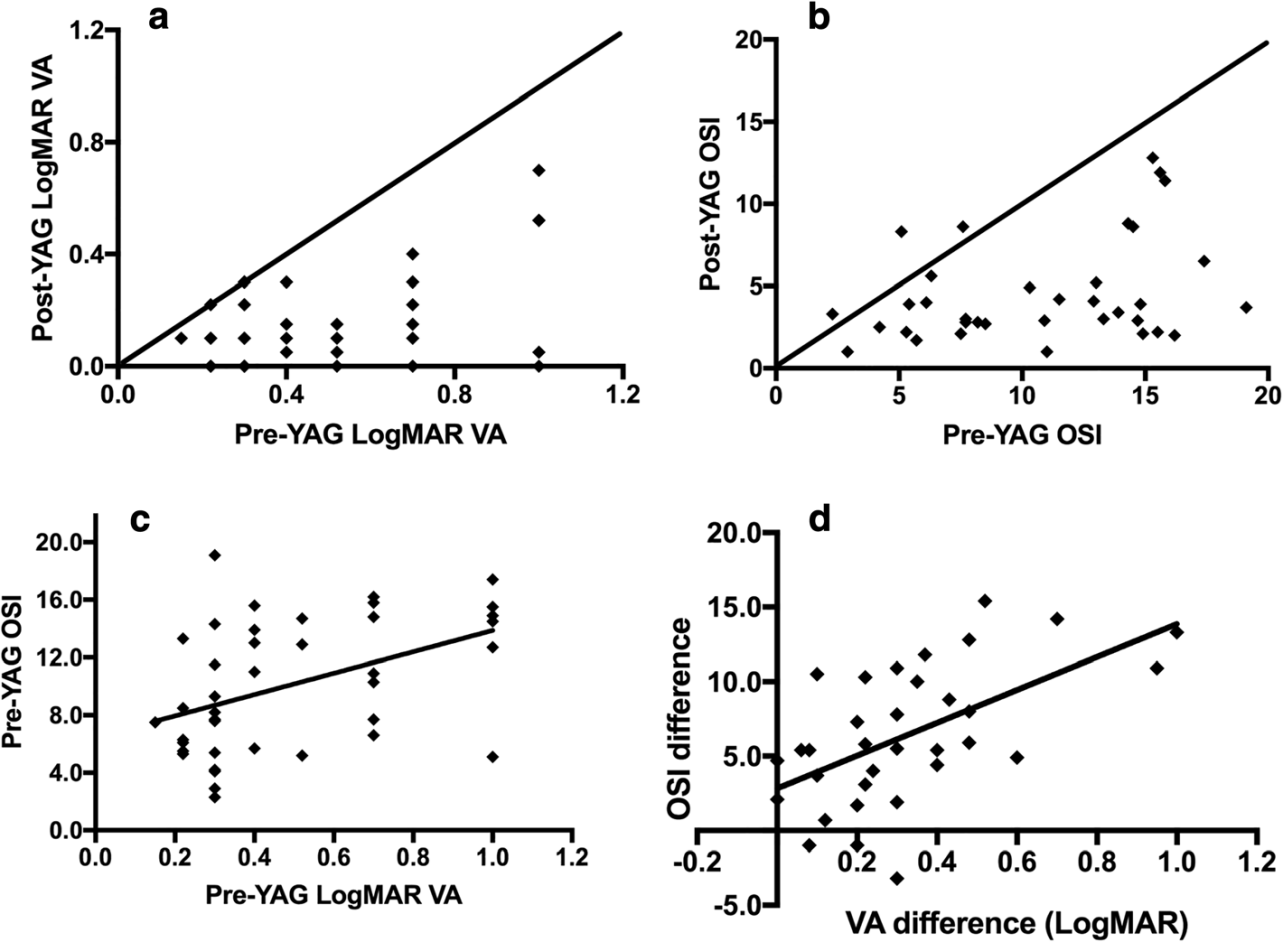
Relationship between VA and OSI. **a** Correlation of VA pre-YAG & post-YAG, **b** correlation of OSI pre-YAG & post-YAG, **c** correlation of VA and OSI before laser capsulotomy, **d** correlation of OSI and VA changes after laser capsulotomy

The LogMAR VA and OSI of PCO patients before and after laser capsulotomy are shown in Table 1. There were significant differences between pre-YAG and post-YAG of LogMAR VA and OSI for all PCO eyes (Table 1, Fig. 2a, b). The pre-YAG LogMAR VA was correlated with the OSI as described in Fig. 2c, and the correlation coefficient was 0.49 (*p* = 0.001). The VA improvement was also positively related to the OSI decrease, described in Fig. 2d, and the correlation coefficient was 0.67 (*p* ≤ 0.001).

**Table 1:** Comparison between LogMAR VA and OSI: Pre-YAG and post-YAG

Total	LogMAR VA	OSI
Pre-YAG	0.50 ± 0.27 (0.15–1.00)	9.87 ± 4.73 (2.30–19.10)
Post-YAG	0.17 ± 0.17 (0.00–0.70)	4.61 ± 3.16 (1.00–12.80)
Difference	0.32 ± 0.24 (0.00–1.00)	6.03 ± 4.63 (− 3.20–15.40)
Z	− 5.16	− 4.83
P	≤0.01	≤0.01

*VA* visual acuity, *OSI* objective scatter index

### Comparison of morphology and severity between pear PCO and fibrosis PCO

**Fig. 3:**
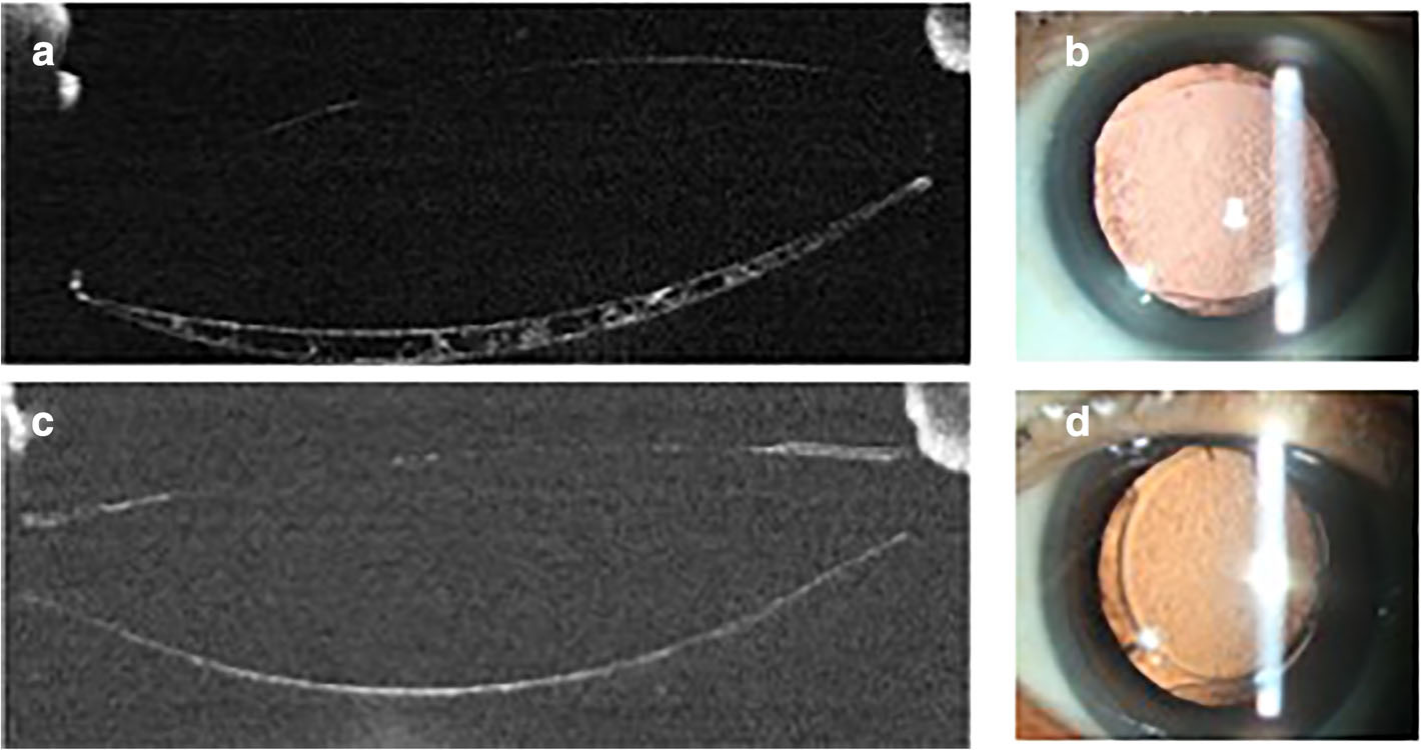
Morphology of pear type and fibrosis PCO. **a** Represents the pear-type PCO using RTVue-100 OCT, and the unevenly distributed opacification was observed in the IOL-posterior capsule space. Parallel retro-illumination image is shown in figure **b**; **c** represents the fibrosis PCO using RTVue-100 OCT, and the strengthened posterior capsule was observed. **d** Retro-illumination image of fibrosis PCO

The arc shaped PCO region ‘DFHIGE’, as described in Fig. 1a, was the central 3-mm PCO region. As shown in Fig. 1b, the IOL outline and the IOL-posterior capsule space of the pseudophakic eye were visualized in the RTVue-100 OCT cross-section images. Pear type PCO (Fig. 3a) was described as the high-density deposition of the proliferated LECs and the extracellular matrix between the IOL and the posterior capsule, which were unevenly distributed, and bladder cells were also observed. Fibrosis PCO (Fig. 3c) was described as the high density of the posterior capsule.

**Fig. 4:**
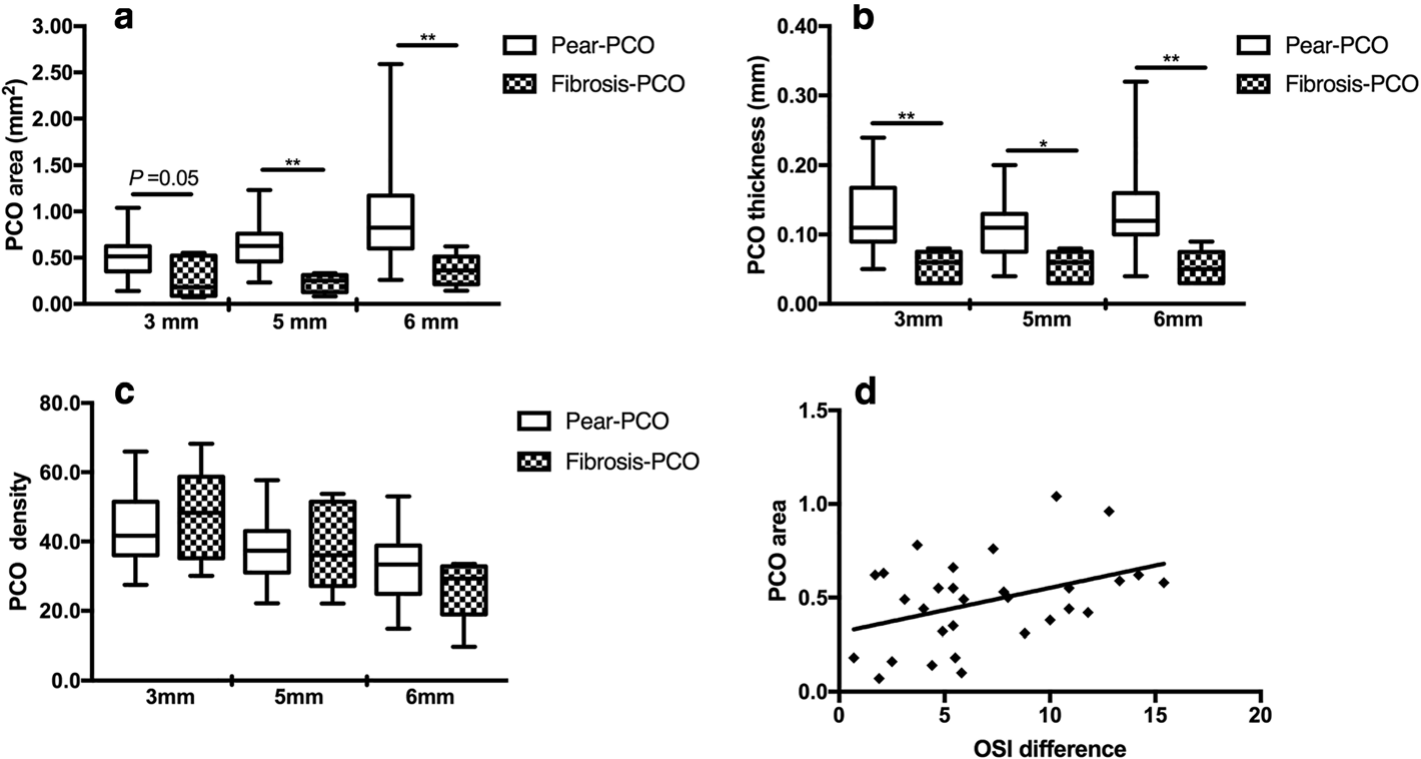
Evaluation of PCO severity using RTVue-100 OCT. **a** Comparison of opacification area between pear type and fibrosis PCO (Mann-Whitney U test, Z = − 1.96, *p* = 0.05 at 3 mm optic zone; ^**^Z = − 3.14, *p* = 0.002 at 5 mm optic zone; ^**^Z = − 2.71, *p* = 0.007 at 6 mm optic zone). **b** Comparison of PCO thickness between pear type and fibrosis PCO, the difference was statistically significant (Mann-Whitney U test, Z = − 3.05, p = 0.002 at 3 mm optic zone; Z = − 2.52, *p* = 0.01 at 5 mm optic zone; Z = − 3.15, p = 0.002 at 6 mm optic zone). **c** Comparison of PCO density between pear type and fibrosis PCO (Mann-Whitney U test, Z = − 0.65, *p* = 0.51 at 3 mm optic zone; Z = − 0.15, *p* = 0.88 at 5 mm optic zone; Z = − 1.00, *p* = 0.32 at 6 mm optic zone). **d** Correlation of OSI changes and PCO area at 3 mm IOL optic region before laser capsulotomy for all eyes (r = 0.43, *p* = 0.02)

The severity of PCO was evaluated with PCO area, thickness and density, which were 0.48 ± 0.24 (0.07–1.04) mm^2^, 0.12 ± 0.06 (0.03–0.24) mm and 44.14 ± 11.09 (27.49–68.26) at 3 mm optic zone, respectively. At 5 mm optic zone, PCO area was area, thickness and density were 0.59 ± 0.31 (0.08–1.23) mm^2^, 0.10 ± 0.05 (0.01–0.20) mm and 39.22 ± 10.61 (22.13–65.3), respectively. PCO area at 3 mm optic zone was significantly correlated with the OSI reduction after laser capsulotomy (r = 0.43, *p* = 0.02) as described in Fig. 4d. While at the 5 mm optic zone, PCO area was not correlated with the OSI decreasing (r = 0.06, *p* = 0.76). The density and thickness were not statistically correlated.

The differences between pear type PCO and fibrosis PCO are shown in Fig. 4a, b, c. The pear type PCO showed a larger opacification area in the IOL-posterior capsule space (Z = − 1.96, *p* = 0.05 at 3 mm optic zone; Z = − 3.14, *p* = 0.002 at 5 mm optic zone; Z = − 2.71, *p* = 0.007 at 6 mm optic zone) and was thicker than the fibrosis PCO (Z = − 3.05, p = 0.002 at 3 mm optic zone; Z = − 2.52, *p* = 0.01 at 5 mm optic zone; Z = − 3.15, p = 0.002 at 6 mm optic zone). The density of these two kinds of PCO was not significantly different (Z = − 0.65, *p* = 0.51 at 3 mm optic zone; Z = − 0.15, *p* = 0.88 at 5 mm optic zone; Z = − 1.00, *p* = 0.32 at 6 mm optic zone). In addition, the area of pear type PCO at 3 mm optic region was correlated with the OSI reduction after laser capsulotomy (r = 0.36, *p* = 0.07); for fibrosis PCO, the correlation decreased (r = 0.32, *p* = 0.6).

### Visual function comparison between pear PCO and fibrosis PCO

Before laser capsulotomy, the pear type PCO showed poor VA compared with fibrosis PCO (Z = − 3.36, *p* = 0.001). After laser capsulotomy, VA increased, and there was no significant difference between the fibrosis PCO and the pear type PCO (Z = − 1.42, *p* = 0.16) (Table 2),

**Table 2:** Comparison between fibrosis PCO and pear type PCO: Pre-YAG and post-YAG

	Fibrosis-PCO	Pear type-PCO	Z	P
LogMAR VA				
Pre-YAG	0.29 ± 0.08 (0.22–0.52)	0.59 ± 0.28 (0.15–1.00)	− 3.36	≤0.01
Post-YAG	0.11 ± 0.11 (0.00–0.30)	0.20 ± 0.19 (0.00–0.70)	− 1.42	0.16
Difference	0.18 ± 0.14 (0.00–0.48)	0.39 ± 0.24 (0.00–1.00)	–	–
Z	−2.81	−4.38	–	–
P	≤0.01	≤0.01	–	–
OSI				
Pre-YAG	5.72 ± 2.08 (2.30–9.30)	11.93 ± 4.13 (4.0–19.10)	−4.06	≤0.01
Post-YAG	3.76 ± 2.36 (1.00–8.60)	4.87 ± 3.37 (1.00–12.80)	−0.71	0.48
Difference	2.05 ± 2.60 (−1.00–5.80)	7.25 ± 4.44 (−3.20–15.40)	–	–
Z	−1.82	−4.36	–	–
P	0.07	≤0.01	–	–

*VA* visual acuity, *OSI* objective scatter index

Pear type PCO showed higher OSI compared with the fibrosis PCO both pre- and post-YAG (Z = − 4.06, *p* ≤ 0.001). Laser capsulotomy significantly decreased the OSI of pear type PCO. The OSI of fibrosis PCO also decreased, though it was not statistically significant. After laser capsulotomy, there was no significant difference in OSI between fibrosis PCO and pear type PCO (Z = − 0.71, *p* = 0.48) (Table 2).

**Fig. 5:**
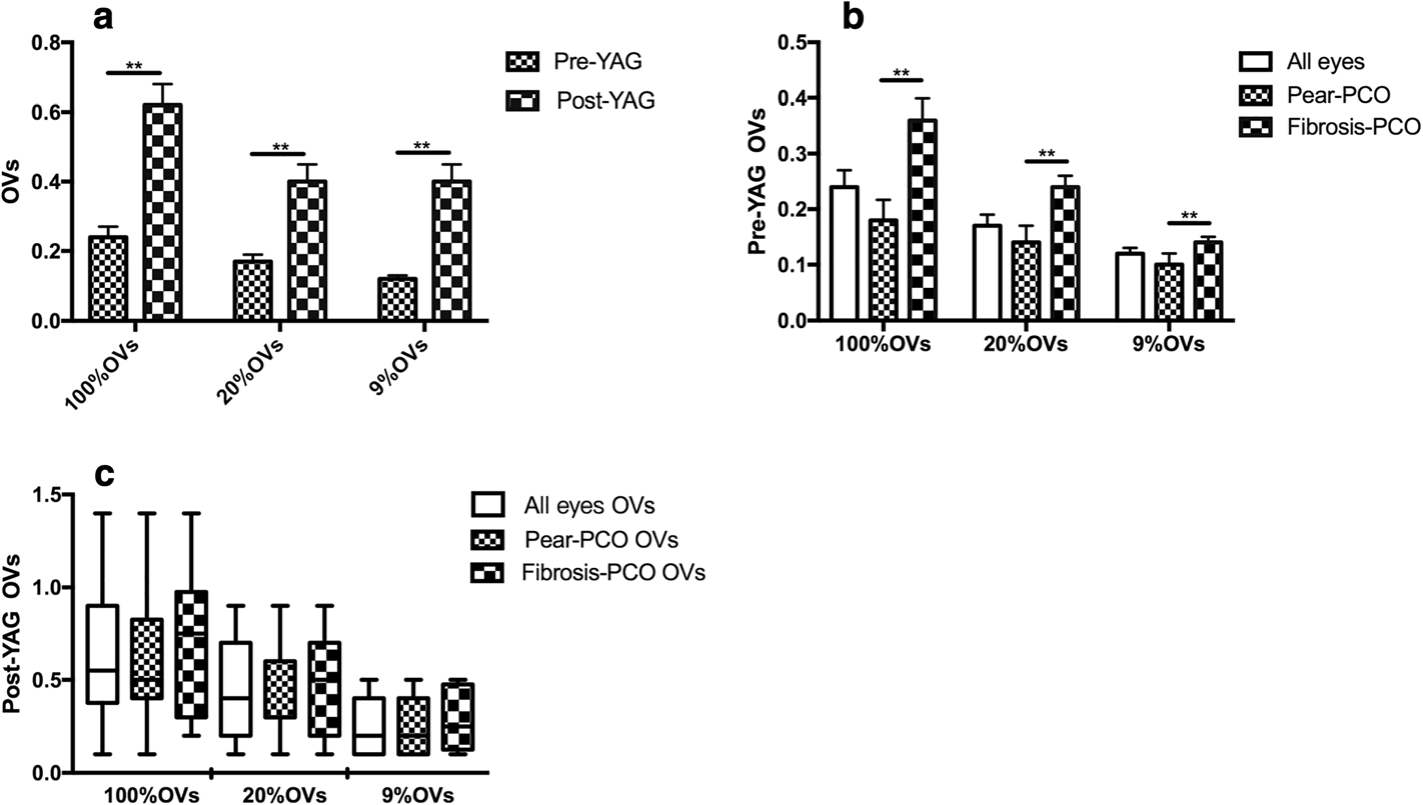
Comparison of OVs values between pear type and fibrosis PCO. **a** 100% OVs, 20% OVs, 9% OVs comparison between pre-YAG and post-YAG, OVs at the three contrast were significantly improved after laser capsulotomy (Mann-Whitney U test, ^**^Z = − 4.31, *p* ≤ 0.001; ^**^Z = − 3.9, p ≤ 0.001; ^**^Z = − 3.96, p ≤ 0.001), **b** 100% OVs, 20% OVs, 9% OVs comparison between pear type PCO and fibrosis PCO before laser capsulotomy, OVs in fibrosis PCO was significantly higher than pear type PCO (Mann-Whitney U test, **Z = − 4.4, p ≤ 0.001; ^**^Z = − 4.34, p ≤ 0.001; ^**^Z = − 3.12, p = 0.002), **c** 100% OVs, 20% OVs, 9% OVs comparison between pear type PCO and fibrosis PCO after laser capsulotomy, there was no significant difference here

Before laser capsulotomy, the 100% OVs, 20% OVs and 9% OVs were 0.24 ± 0.20, 0.18 ± 0.13, and 0.12 ± 0.09, respectively, which significantly improved after laser capsulotomy: 0.62 ± 0.36, 0.39 ± 0.28 and 0.25 ± 0.15, respectively (Fig. 5a). The improvements were statistically significant (Z = − 4.31, *p* ≤ 0.001; Z = − 3.9, p ≤ 0.001; Z = − 3.96, *p* ≤ 0.001).

Comparing the OVs of fibrosis and pear type PCO, similar improvements were observed (in Fig. 5b, c). However, fibrosis PCO showed higher OVs than the pear type PCO both pre-YAG and post-YAG. In addition, there were significant differences in the 100% OVs, 20% OVs and 9% OVs between fibrosis and pear type PCO before laser capsulotomy (Z = − 4.4, p ≤ 0.001; Z = − 4.34, *p* ≤ 0.001; Z = − 3.12, *p* = 0.002). After laser capsulotomy, the differences were not statistically significant.

### The relationship between OSI and 100% OVs, 20% OVs and 9% OVs

The OVs evaluated the objective visual acuity under three contrast conditions, as described in Table 3. Before laser capsulotomy, there were correlations between OVs and OSI both in pear type PCO and fibrosis PCO at 100 and 20% contrast levels, except for the low contrast level of 9%. After laser capsulotomy, OSI was significantly correlated with OVs at all the contrast levels in pear type PCO but was not significantly correlated with that of fibrosis PCO.

**Table 3:** Relationship between OSI and 100% OVs, 20% OVs and 9% OVs

	OSI & 100% OVs		OSI & 20% OVs		OSI & 9% OVs	
	correlation coefficient	*P* value	correlation coefficient	*P* value	correlation coefficient	*P* value
Fibrosis-PCO						
Pre-YAG	−0.64	0.02	−0.61	0.02	−0.36	0.21
Post-YAG	−0.67	0.07	−0.69	0.06	−0.52	0.19
Pear type-PCO						
Pre-YAG	−0.67	0.00	−0.62	0.00	−0.07	0.74
Post-YAG	−0.42	0.03	−0.45	0.02	−0.48	0.01

OSI, objective scatter index. OVs, optical quality analysis system values

## Discussion

Visual function does not decline substantially until 50 years of age, but especially declines in the age range of 61–70 years [19]. Stray light increases strongly with age^3^ and doubles by the age of 65 years, tripling by the age of 77 years [20]. PCO is heterogeneous both in morphology and severity, and stray light increases in PCO eyes, which is considered an early indicator [16].

In our study, OQAS-II was used to objectively evaluate the intraocular stay light. What we found was that pre-YAG OSI was 9.87 ± 4.73 and that LogMAR VA was 0.50 ± 0.27. It was reported that in the pseudophakic eye, OSI was 1.06 ± 0.48 and LogMAR VA was − 0.26 ± 0.06; in normal eyes, OSI was 1.03 ± 0.65 and LogMAR VA was − 0.25 ± 0.06 [21]. Our finding supports the points that PCO induced visual function deterioration, including VA reduction and stray light increases [22–27]. In addition, we found that increased OSI was correlated with decreased visual acuity. As previously reported, the more light that was scattered, the more VA and CS decreased in PCO eyes [24]. Zhang’s study [11] also proved the close relationship between OSI and BCVA in PCO eyes, and their correlation coefficient was higher than ours. This discrepancy may be explained by the uneven distribution and severity of different kinds of PCO, and the visual acuity and stray light of PCO eyes did not correspond [22].

After laser capsulotomy, OSI decreased obviously, accompanied by significant VA improvement. The changes in OSI were well correlated with those of VA. These findings are supported by previous studies showing that laser capsulotomy removed the opacified posterior capsule and increased VA, CS [24–27] and intraocular stay light values [8, 26]. Yotsukura found that the log(s) of the stray light values significantly decreased after capsulotomy (pre-YAG 1.59 ± 0.20 log(s) & post-YAG 1.43 ± 0.14 log(s)) [26]. Nino reported that the OSI of regenerative PCO pre-YAG was 8.0 ± 4.6 and was 3.6 ± 2.2 post-YAG; meanwhile, the stray light measured with C-quant was 1.8 ± 0.6 log (s) pre-YAG and 1.6 ± 0.3 log(s) post-YAG. The log(s) of stray light was correlated with OSI (r = 0.32, *p* = 0.07) [8]. Similar changes occurred in cataract eyes. OSI was 11.5 ± 3.6 pre-surgery, which significantly decreased (3.2 ± 0.8) with OSI 2 months after cataract surgery, accompanied by the improvement of BCVA [28].

To assess the effect of PCO morphology and severity on visual function, we classified the fibrosis and pear type PCO and analyzed the area, thickness and density [16] of these two kinds of PCO using RTVue-100 OCT.

We found a positive correlation between the 3-mm PCO area and the OSI difference, which suggests that the larger opacification area might be paralleled by higher scatter light. We define PCO severity by multiplying the PCO area by density and found that it was also related with the OSI but not with the VA. This finding agreed with the previous finding that in PCO patients, stray light increased with good visual acuity [26]. PCO severity evaluated with the EPCO score had a liner relationship with the log of the stray light parameters s (log(s)) and a curvilinear relationship with log MAR VA. VA and stray light were two independent factors, and the increase in stray light was much more sensitive than the decrease in VA [16]. Furthermore, PCO’s effect on the small angle or large angle domain visual function may depend on the ratio of small particles and the refractile structures of PCO [23].

When comparing the pear type PCO with fibrosis PCO, we found a larger area and a thicker sub-capsular opacification but a higher density of fibrosis PCO than pear type PCO. As reported, fibrosis PCO originated from cuboidal epithelial cells that lined the anterior capsule and resulted in wrinkling and thickening of the adjacent posterior capsule. It was reported that EPCO scores and PCO refractions evaluated with retro-illuminations in pear type PCO were significantly higher than those of the fibrosis PCO [16]. Pear type PCO primarily originated from the actively mitotic epithelial cells located at the lens equator, which was the origin from which the bladder cells tended to migrate to form posterior sup-capsule opacification. Different origins and pathological processes may explain the different characteristics of fibrosis and pear type PCO [24].

In addition, Romina et al correlated the PCO severity with visual function and suggested that clinically considered high density fibrosis PCO might have a less deleterious effect on visual function compared with less severe pear PCO because light may be attenuated by the dense fibrosis PCO but may be scattered more in the pear PCO [29]. Our result agreed with the above study that the straticulate and high-density fibrosis PCO showed lower OSI (5.72 ± 2.08 &11.93 ± 4.13) and higher VA (0.29 ± 0.08 & 0.59 ± 0.28) compared with pear type PCO. Maartje also reported higher log(s) in pear type PCO than fibrosis PCO [16]. After laser capsulotomy, there were no significant differences between fibrosis and pear type PCO both in VA and stray light. Previous in vitro studies supported the varied visual function in pear type PCO and fibrosis PCO, and the author analyzed the forward light scatter using different kinds of posterior capsule opacification and suggested that small particles, rod-like fibers in fibrosis PCO, and pear-like structures in regenerative PCO were related to the decrease in VA and the increase in stray light [22].

Since the daily optical environment varied in the light contrast, VA did not reflect visual function comprehensively. We evaluated the VA under three contrast conditions, 100, 20, and 9%. The OVs in our study were lower than those of the pseudophakic eyes [10, 12], and the author suggested that OVs in pseudophakic eyes were similar or superior to those in normal eyes [12]. PCO deteriorated visual function at all the contrast levels, and OVs decreased when the contrast level was reduced. Similar results were reported using OQAS II that simulated CS were 35.5 + 27.8 at 100%, 32.9+ 24.2 at 20%, and 32.4+ 21.3 at 9% pre-laser capsulotomy and that they improved to 62.7 + 33.4 at 100%, 57.6 + 29.2 at 20%, and 55.0 + 24.2 at 9% post-laser capsulotomy. Fibrosis PCO showed significantly higher OVs than pear type PCO at all the contrast levels, which was in line with the report that pear type PCO lost CS at all frequencies, which was significantly worse than that of fibrosis PCO [30]. In addition, the decreased OVs were correlated with the increase in OSI both in the fibrosis PCO and pear type PCO with the 100 and 20% contrast levels but not with the low contrast level of 9%.

Our study has some limitations. The number of fibrosis PCOs was small, which may have limited the fibrosis PCO results. However, the results of fibrosis PCO in our study were consistent with previous results as we discussed above. Another limitation was that we used RTVue-100 OCT to assess the PCO severity on two cross-sectional images of the anterior segment using the opacification area, thickness and density. We found differences between fibrosis PCO and pear type PCO, and the correlation between PCO area with OSI changed, but we could not ignore the fact that cross-sectional images cannot represent the complete opacification. Therefore, further study with a more comprehensive method may be needed.

In summary, PCO caused visual function impairments including increased scattered light and decreased VA. The increased intraocular scattered light was significantly correlated with the decrease in VA and the decrease in OVs. Pear type PCO tended to result in more serious visual impairment compared with fibrosis PCO, such as the lower VA and OVs with the 100, 20, and 9% contrast levels, as well as the higher OSI. This result agreed with the morphology and severity of pear type PCO, which tended to show a thicker and wider opacification compared with the fibrosis type.

## Conclusion

PCO with different morphology and severity showed different degrees of light scattering and objective visual function impairment. Laser capsulotomy removed PCO and improved visual function. OQASII measured OSI and OVs could objectively indicate the visual function impairment caused by PCO.

## Abbreviations

CDVA: Corrected visual acuity
CS: Contrast sensitivity
logMAR: Logarithm of the minimum angle of resolution
MTF: Modulation transfer function
Nd:YAG: Yttrium Aluminum Garnet
OQAS II: Optical Quality Analysis System II
OSI: Objective scatter index
OVs: Optical quality analysis system values
PCO: Posterior capsule opacification
VA: Visual acuity
